# Risk factors for non-communicable diseases related to obesity among first- and second-generation Bangladeshi migrants living in north-east or south-east England

**DOI:** 10.1038/s41366-021-00822-5

**Published:** 2021-05-04

**Authors:** N. Akhter, K. Begum, P. Nahar, G. Cooper, D. Vallis, A. Kasim, G. R. Bentley

**Affiliations:** 1 Department of Anthropology, Durham University, Durham, UK; 2 Fuse–UKCRC Centre for Translational Research in Public Health, Newcastle upon Tyne, UK; 3 Queen Mary University, London, UK; 4 Department of Global Health and Infection, University of Sussex, Brighton, UK; 5 Durham Research Methods Centre, Durham University, Durham, UK

**Keywords:** Risk factors, Epidemiology

## Abstract

**Background:**

Obesity is a global burden, which significantly increases the risk of non-communicable diseases (NCDs). More than a quarter of adults in the United Kingdom are obese, but prevalence varies by ethnicity, and South Asians have the largest burden of NCDs. This paper assesses how sex, generation, and region interplay to vary the predisposition to obesity-related (OR) NCDs among UK Bangladeshis.

**Methods:**

We used National Institute for Health and Care Excellence suggested grading for combining body mass index and waist circumference to define populations at risk of OR-NCDs. Data from 517 adults of Bangladeshi origin from a cross-sectional study (March 2013 to April 2015) were analysed. Male and female participants from London and north-east England were equally sampled including: (1) adult migrants, who came to the UK aged >16 years; (2) child migrants, who came to the UK aged ≤16 years; and (3) second-generation Bangladeshis (who were born and brought up in the UK). A generalised estimating equation using a binomial distribution and a logit link was used to explore the relationship between the binary outcome of being ‘at risk of OR-NCDs’ and associated factors.

**Results:**

Females, married individuals, those living in London, the second-generation, and those of lower self-assessed financial status, with low acculturation status, or who did not walk daily for at least 20 min were more likely to develop OR-NCDs. A striking sex difference was found with more females prone to OR-NCD risk in the north-east than in London.

**Conclusions:**

Our study observed important inter- and intra-regional inequality in OR-NCD risk which worsens the health of ethnic minorities and widens inequality.

## Introduction

Obesity, ranked as the sixth leading cause of worldwide burden of disease [1], is a multi-dimensional phenomenon, including physiological [2–4], psychological [5, 6], cultural [7, 8], behavioural [8, 9] and environmental factors [10, 11]. It significantly increases the risk of developing non-communicable diseases (NCDs) such as diabetes, hypertension, stroke and cardiovascular diseases (CVD) [1, 12, 13]. Worldwide, about 1.9 billion adults were overweight in 2016, including >650 million obese [14]. The UK, with >25% of adults with obesity, has the sixth highest prevalence of adult obesity among countries in the Organisation for Economic Co-operation and Development [15]. In England, about 35% of adults are estimated to be obese by 2030 [15]. Obesity causes >30,000 deaths annually in England, reducing life expectancy by 9 years [16]. The increased prevalence of overweight and obesity in the UK costs the National Health Service (NHS), and tax payers, an estimated £27 billion per annum [16]. Reducing the prevalence of overweight and obesity is a high priority for Public Health England [16], while tackling obesity-related, non-communicable diseases (OR-NCDs) remains an important goal in reducing health inequalities [4].

The burden of obesity and overweight together with associated diseases poses specific challenges for ethnic minorities [17]. South Asians are at higher risk for developing OR-NCDs and have significantly lower levels of physical activity than white Europeans [4, 9]. However, since each South Asian group has distinct characteristics (including Bangladeshis, Indians and Pakistanis), over-arching categorisations can be simplistic. Within South Asian countries, including Bangladesh, the prevalence of overweight and obesity is also rising [18], with an increase in undiagnosed Type 2 diabetes mellitus (T2DM) and pre-diabetic conditions [19]. However, compared with white European and other ethnic groups, the prevalence of obesity is higher among the UK migrant, Bangladeshi population [3]. Moreover, the risk of CVD was 70% and 30% higher among immigrant Bangladeshi women and men, respectively, than white or other local residents in Europe [9]. Particular attenditon to health needs is therefore required for UK Bangladeshis who have high fat diets [20], low levels of physical activity [7], and the highest standardised CVD-related mortality rates among ethnic minority groups [4], with high fat diets [20] and low levels of physical activity [7].

Although several authors have documented health inequalities among ethnic minorities, further understanding is needed concerning the interplay of biological, genetic, developmental, socioeconomic, demographic, religious, cultural and behavioural factors [21–27]. For example, Higgins et al. demonstrated a strong association between waist circumference (WC) and ethnicity in the UK, with WC increasing in proportion to migrants’ length of residence [3]. Apart from a potential biological predisposition to obesity [2], poorer socioeconomic conditions among ethnic minorities is associated with higher risk of OR-NCDs [3, 23]. While obesity is also more prevalent among people living in deprived areas [11], poverty and area-level deprivation increase health inequalities [28], and Bangladeshis tend to be concentrated in the most deprived areas of the UK [3].

This cross-sectional study aims to assess how sex, generation and region are associated with OR-NCDs in Bangladeshis living in either north-east (NE) England or London, using indicators of health risk that combine the body mass index (BMI) and WC. The paper aims to support academics, health professionals and policy makers in responding to ethnic inequalities, and in designing effective policies to reduce morbidity. We used the National Institute for Health and Care Excellence (NICE) grading to define populations at risk (increased/high/very high) of developing OR-NCDs [29]. Given that obesity and OR-NCDs are more prevalent among female Bangladeshis [18]; that first-generation adult migrants undertake very little physical activity [7]; and NE England is generally more deprived than London [30], we hypothesised that OR-NCD risk would be higher among women, first-generation adult migrants in the NE, and those of poor socioeconomic status (SES).

## Methods

### Subjects

We collected data between March 2013 and April 2015 from 562 males and females of Bangladeshi origin, aged 25–40 years. Participants from London and NE England were sampled equally within each region, and further divided into: (1) first-generation, adult migrants (who came to the UK aged >16 years); (2) first-generation child migrants (who came to the UK aged ≤16 years); and (3) second-generation, British-Bangladeshis (born and raised in the UK). Data were collected through face-to-face administration of a structured questionnaire. Participants were recruited through local contacts and snowballing in five London boroughs with large numbers of Bangladeshis: Tower Hamlets, Camden, Redbridge, Newham, Barking and Dagenham. In NE England, residents from Darlington, Newcastle, North Shields, Hartlepool, South Shields, Sunderland and nearby areas were included.

### Exclusion criteria

Due to effects on weight and metabolism, pregnant and lactating women were excluded, as were participants clinically diagnosed with psychosis, bipolar disorder, severe depression, thyroid conditions or Type 1 diabetes. Due to some missing information (45 subjects), data from a sample of 517 subjects were analysed.

### Study tools

Participants answered a 127-item, structured questionnaire concerning demographic history, socioeconomic and educational background, issues of acculturation, health histories, dietary intake and physical exercise. The questionnaire and anthropometric assessments took ~1.5 h (on average) to complete. The former included standardised questionnaires: the 12-item General Health Questionnaire (GHQ-12) [31], an adapted Suinn-Lew Asian Self-Identity Acculturation Scale [32], and the Everyday Discrimination Scale [33]; the questionnaire was translated into Bangla, and independently back-translated into English to check for accuracy. Draft questionnaires were piloted extensively and modified following feedback. The participants were given the option of a Bangla or English version. Few participants (12 in London; 2 in the NE) chose the written Bangla questionnaire. Most interviews were conducted in either Bangla or the Sylheti dialect, particularly with first-generation migrants, although responses were written in English.

Acculturation score, averaged responses about participants’ language proficiency and preferences, pride as Bangladeshis, strength of religious traditions, participation in Bangladeshi festivals, ethnicity of friends, frequency of visits to Bangladesh, perception of fitting well within different ethnic groups, preference for types of book, media, food and clothing, and dining with Bangladeshi and non-Bangladeshi families, ranged from 1 (least acculturated) to 5 (most acculturated). Using tertile cut-offs, the score was converted to three categories: low (<2.45), medium (2.45–2.9) and high (>2.91). Similarly, a discrimination index was calculated from the Everyday Discrimination Scale. A higher score reflected more frequent or intense experiences of discrimination.

### Anthropometry

Anthropometric data were collected using standardised techniques [34], including height, weight, mid-upper arm circumference (MUAC), hip circumference and WC. Height was measured using a portable stadiometer to the nearest millimetre. A Seca scale was used to measure weight to the nearest 100 g, and a flexible Seca tape was used to measure WC, hip circumference and MUAC to the nearest millimetre. The BMI was calculated as weight(kg)/height(m)^2^. World Health Organization (WHO) classifications for underweight (<18.5 kg/m^2^), normal (18.5–24.9 kg/m^2^), overweight (25.0–29.9 kg/m^2^) and obese ≥30 kg/m^2^ were used [14]. The BMI-based obesity assessment reflects generalised rather than abdominal obesity, the latter estimated using WC [35]. Sex-specific cut-offs for high WC (men: >94 cm and women: >80 cm) were used [36].

Abdominal obesity reflects a large amount of intra-abdominal fat including visceral adipose tissue and is considered a better measure of body fat and associated morbidities [13, 36]. BMI is less accurate in measuring adiposity, and the conventional obesity cut-offs points may underestimate NCD prevalence in South Asians [2, 13, 27, 36, 37]. Its weakness as a predictor of insulin resistance, T2DM and CVD risk factors in South Asians and their migrant compatriots has been well documented [2, 27, 36, 38]. Higher centripetal fat among South Asians is associated with higher susceptibility to NCDs at a BMI ≤ 30 kg/m^2^ [24]. Those with a ‘normal’ BMI, but large WC, may have a two- to threefold higher risk of CVD and premature death [35]. The WHO therefore recommends using WC alongside BMI as an obesity measure [39]. NICE has suggested interpreting BMI cautiously, and to assess health risk by combining both BMI and WC [29, 36].

### Statistical analyses

Data were summarised as means and standard deviations for continuous data, and percentages for categorical data. Combining NICE categories for increased, high and very high health risks (Supplementary Table 1), a binary outcome of being ‘predisposed to OR-NCDs’ was created. Analyses were done in SAS version 9.4. A generalised estimating equation with a binomial distribution and a logit link was used to explore the relationship between the outcome and covariates. The analyses also accounted for clustering of participants within boroughs. Initial analyses examined bivariate associations between being ‘at risk of chronic disease’, and relevant predictors. Predictors included: (1) sex (male, female); (2) region (NE, south); (3) generation (first-generation adult migrants, first-generation child migrants, second-generation); and (4) age (years). Health-related covariates were: (1) the GHQ-12 following the Likert method (0–3) [31], (2) self-reported health status (poor, fair, good, very good, excellent) and (3) involvement in physical activity (none, limited, most-days/regular). Predictors of social and cultural status included: (1) acculturation (low, medium, high); (2) education (GCSE or equivalent, A-level or equivalent, undergraduate, postgraduate); (3) self-rated, current financial status (struggling, okay, comfortable/well-off); (4) employment (unemployed, employed, homemaker/voluntarily unemployed); and (5) discrimination status (experience of day-to-day discrimination). Other variables included in the model were: a continuous score for social support (using the MOS Social Support survey) [40], self-reported diet (intake of fruits, vegetables, fizzy drinks and take-away foods), self-reported physical activity (walking ≥ 20 min daily) and participation in sports (daily sports/exercise). Variables with potential conceptual and statistical associations were included in the covariate-adjusted model. Age, sex and region have known associations with health [17], and were included in the model as potential confounders between the outcome and socio-cultural factors (Supplementary Table 2). The final parsimonious model was obtained by backward selection, where larger *p* values were sequentially left out starting with interaction terms. Sensitivity analyses for missing data used multiple imputations with chained equations, and a ten-fold cross-validation was used to assess prediction accuracy (Supplementary Fig. 2) of the model.

## Results

Descriptive variables are shown in Tables 1 and 2. The percentages of female and male participants were equally distributed among the first-generation, child migrants and second-generation adults. About 31% of participants were aged 25–29 years, 29% were 30–34 years and 40% were 35–40 years. Compared to first-generation adults or child migrants (both 22%), more second-generation individuals were aged <30 years (50%) reflecting the history of migration to the UK. Median length of stay in the UK for all participants was 25 years (25th percentile: 13 years; 75th percentile: 30 years), and was similar in both north and south. Overall, most participants were married (80%), while 15% was single and 5% separated, or divorced. However, almost double the proportion of first-generation child migrants and the second-generation were separated or divorced compared to first-generation, adult migrants. As expected from the comparatively younger second-generation, a higher proportion (26%) was single compared to adult/child migrants (both 9%). Roughly one-third of participants had up to A-Level or equivalent education, and about a quarter had completed higher education. Compared to adult migrants (29%), a larger proportion of child migrants (84%) and second-generation individuals (69%) had completed higher education.

**Table 1:** Descriptive data for 517 Bangladeshis living in north-east (*n* = 253) and southern England (London, *n* = 264).

Variables	London	North-east	Overall	*p* value
	% (*n*)	% (*n*)	% (*n*)	
Sex				
Male	47.7 (126)	49.8 (126)	48.7 (252)	0.637
Female	52.3 (138)	50.2 (127)	51.3 (265)	
Generations				
1st Adult	34.1 (90)	33.6 (85)	33.8 (175)	0.910
1st Child	30.7 (81)	32.4 (82)	31.5 (163)	
2nd Generation	35.2 (93)	34.0 (86)	34.6 (179)	
Age				
23–29 years	29.2 (77)	33.6 (85)	31.3 (162)	0.209
30–34 years	27.3 (72)	30.4 (77)	28.8 (149)	
35 and above	43.6 (115)	36.0 (91)	39.8 (206)	
Length of stay				
<10 years	13.3 (35)	15.0 (38)	14.1 (73)	0.820
10–19 years	23.9 (63)	21.7 (55)	22.8 (118)	
20–29 years	35.6 (94)	37.9 (96)	36.8 (190)	
30 and above years	27.3 (72)	25.3 (64)	26.3 (136)	
Marital status				
Single	15.5 (41)	13.8 (35)	14.7 (76)	0.301
Married	80.7 (213)	79.4 (201)	80.1 (414)	
Separated/Divorced	3.8 (10)	6.7 (17)	5.2 (27)	
Education				
O-level equivalent	33.0 (87)	36.4 (92)	34.6 (179)	0.032
A-level equivalent	36.7 (97)	44.3 (112)	40.4 (209)	
Undergraduate	18.9 (50)	11.1 (28)	15.1 (78)	
Postgraduate	11.4 (30)	8.3 (21)	9.9 (51)	
Financial status				
Struggling	11.7 (31)	19.4 (49)	15.5 (80)	0.043
Okay	51.7 (137)	50.2 (127)	51.1 (264)	
Comfortable/Well-off	36.4 (96)	30.4 (77)	33.5 (173)	
Employment				
Employed	64.0 (169)	62.8 (159)	63.4 (328)	<0.001
Unemployed	6.8 (18)	19.4 (49)	13.0 (67)	
Homemaker/Voluntary unemployed	29.2 (77)	17.8 (45)	23.6 (122)	
Housing				
Own	6.8 (18)	7.2 (18)	7.0 (36)	<0.001
Mortgage	29.7 (78)	49.6 (124)	39.4 (202)	
Rent	63.5 (167)	43.2 (108)	53.6 (275)	
Acculturation				
Low	25.8 (68)	34.8 (88)	30.2 (156)	0.043
Medium	36.0 (95)	35.6 (90)	35.8 (185)	
High	38.3 (101)	29.6 (75)	34.0 (176)	
Discrimination				
Yes	48.7 (131)	69.7 (161)	58.4 (292)	0.162
No	51.3 (138)	30.3 (70)	41.6 (208)	

All results presented in the table are unadjusted differences.

**Table 2:** Descriptive data for Bangladeshi adult migrants (*n* = 175), child migrants (*n* = 163) and second-generation British-Bangladeshis (*n* = 179) living in north-east and southern England (total *n* = 517).

Variables	Adult migrants	Child migrants	Second generation	Overall	*p* value
	% (*n*)	% (*n*)	% (*n*)	% (*n*)	
Sex					
Male	50.9 (89)	46.6 (76)	48.6 (87)	48.7 (252)	0.738
Female	49.1 (86)	53.4 (87)	51.4 (92)	51.3 (265)	
Age					
23–29 years	21.7 (38)	21.5 (35)	49.7 (98)	31.3 (162)	<0.001
30–34 years	29.1 (51)	24.5 (40)	32.4 (58)	28.8 (149)	
35 and above	49.1 (86)	54.0 (88)	17.9 (32)	39.8 (206)	
Length of stay					
<10 years	41.1 (72)	0.6 (1)	0.0 (0)	14.1 (73)	<0.001
10–19 years	54.9 (96)	13.5 (22)	0.0(0)	22.8 (118)	
20–29 years	4.0 (7)	57.1 (93)	50.3 (90)	36.8 (190)	
≥30 years	0.0 (0)	28.8 (47)	49.7 (89)	26.3 (136)	
Marital status					
Single	9.1 (16)	8.6 (14)	25.7 (46)	14.7 (76)	<0.001
Married	88.0 (154)	85.3 (139)	67.6 (121)	80.1 (414)	
Separated/Divorced	2.9 (5)	6.1 (10)	6.7 (12)	5.2 (27)	
Education					
Up to secondary	41.7 (73)	6.1 (10)	12.3 (22)	9.9 (51)	<0.001
Higher secondary	31.4 (55)	9.8 (16)	19.0 (34)	15.1 (78)	
Undergraduate	16.0 (28)	44.2 (72)	45.8 (82)	40.4 (209)	
Postgraduate	10.9 (19)	39.9 (65)	22.9 (41)	34.6 (179)	
Financial status					
Struggling	18.3 (32)	17.8 (29)	10.6 (19)	15.5 (80)	<0.001
Okay	55.4 (97)	55.2 (90)	43.0 (77)	51.1 (264)	
Comfortable/Well-off	26.3 (46)	27.0 (44)	46.4 (83)	33.5 (173)	
Employment					
Employed	55.4 (97)	63.8 (104)	70.9 (127)	63.4 (328)	0.041
Unemployed	15.4 (27)	11.7 (19)	11.7 (21)	13.0 (67)	
Homemaker/Vol unemployed	29.1 (51)	24.5 (40)	17.3 (31)	23.6 (122)	
Housing					
Own	6.3 (11)	4.3 (7)	10.1 (18)	7.2 (37)	<0.001
Mortgage	23.6 (41)	46.3 (75)	48.3 (86)	39.3 (202)	
Rent	70.1 (122)	49.4 (80)	41.0 (73)	53.5 (275)	
Acculturation					
Low	62.9 (110)	25.2 (41)	2.8 (5)	30.2 (156)	<0.001
Medium	33.1 (58)	46.0 (75)	29.1 (52)	35.8 (185)	
High	4.0 (7)	28.8 (47)	68.2 (122)	34.0 (176)	
Discrimination					
No	50.6 (81)	38.1 (59)	38.4 (66)	42.3 (206)	0.034
Yes	49.4(79)	61.9 (96)	61.6 (106)	57.7 (281)	
Body mass index					
Underweight	2.1 (4)	2.7 (5)	2.7 (5)	2.5 (14)	0.807
Normal	42.0 (79)	43.2 (79)	42.0 (79)	42.4 (237)	
Overweight	43.6 (82)	38.3 (70)	37.8 (71)	39.9 (223)	
Obesity	12.2 (23)	15.8 (29)	17.6 (33)	15.2 (85)	
Waist circumference					
Low	45.1 (79)	47.9 (78)	42.0 (72)	44.3 (229)	0.476
High	25.1 (44)	28.2 (46)	27.9 (50)	27.1 (140)	
Very high	29.7 (52)	23.9 (39)	31.8 (57)	28.6 (148)	

All results presented in the table are unadjusted differences.

For SES, overall, 16% reported they were ‘struggling’ (12% in London and 19% in the NE). More than half of participants (63%) was employed, but the NE had significantly higher unemployment. The unemployed proportion (homemaker or voluntarily unemployed) was highest among first-generation migrants (45%), followed by child migrants (36%) and the second generation (29%). The majority of participants were renters (54%), while <10% were home owners. Renting was common for each group (adult migrants: 70%; child migrants: 49%; second generation: 41%), but twice the number of child migrants (46%) and second generation (48%) were home owners with mortgages compared to adult, first-generation migrants (24%). About 30% of participants had low acculturation scores, while 36% were moderately and 34% highly acculturated. Acculturation was significantly higher in Londoners than those in the NE. Acculturation scores varied markedly with <5% of first-generation, adult migrants having high scores compared to nearly 70% of the second generation. While nearly half of any generation had experienced discrimination in their day-to-day interactions with non-Bangladeshis, scores were much higher in the NE than for London residents (70 vs. 49%).

Table 3 presents health data by sex. Based on self-reported, general health, 16% had poor/fair status, while 42% had very good/excellent general health. However, a large sex difference was present among those reporting excellent health (8% females vs. 22% males). Females had somewhat higher overweight and obesity prevalence than males (59 vs. 51%). Similarly, a much higher proportion of women had a large WC (46% female vs. 10% male). While nearly half of participants reported walking for ≥20 min daily, more females walked daily compared to males (55 vs. 49%), but <10% of both reported participating in sports on a regular basis.

**Table 3:** Health status of UK Bangladeshi male and female participants (*N* = 517).

Indicators	Category	Female	Male	Overall	*p* value
		% (*n*)	% (*n*)	% (*n*)	
General Health	Poor	1.1 (3)	1.2 (3)	1.2 (6)	<0.001
	Fair	9.5 (24)	20.1 (53)	14.9 (77)	
	Good	46.4 (123)	37.3 (94)	42.0 (217)	
	Very good	27.5 (73)	27.0 (68)	27.3 (141)	
	Excellent	7.9 (21)	21.8 (55)	14.7 (76)	
Body mass index	Underweight	1.1 (3)	3.2 (8)	2.3 (11)	0.011
	Normal	40.0 (106)	46.0 (116)	42.9 (222)	
	Overweight	39.6 (105)	40.5 (102)	40.0(207)	
	Obese	19.2 (51)	10.3 (26)	14.9 (77)	
Waist circumference	Low	22.3 (59)	67.5 (170)	44.3 (229)	<0.001
	High	31.7 (84)	22.2 (56)	27.1 (140)	
	Very high	46.0 (122)	10.3 (26)	28.6 (148)	
Physical activity	Not everyday	93.6 (248)	91.3 (230)	92.5 (478)	0.332
	Everyday	6.4 (17)	8.7 (22)	7.5 (39)	
Walk 20 min/day	No	45.3 (120)	50.8 (128)	48.0 (248)	0.210
	Yes	54.7 (145)	49.2 (124)	52.0 (269)	

All results presented in the table are unadjusted differences.

**Fig. 1:**
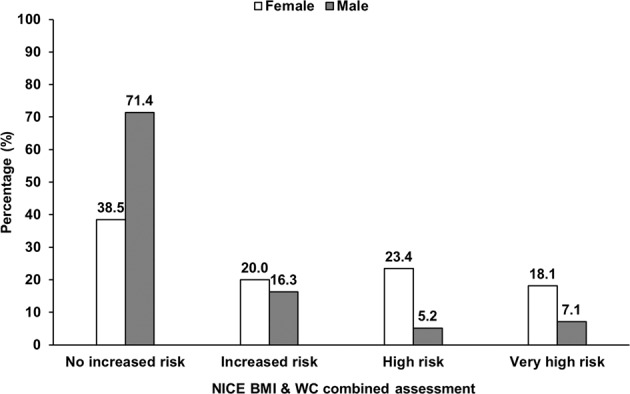
Distribution of study population according to predisposition to OR_NCDS, disaggregated by sex (No colour bars = female, grey bars = male).

**Fig. 2:**
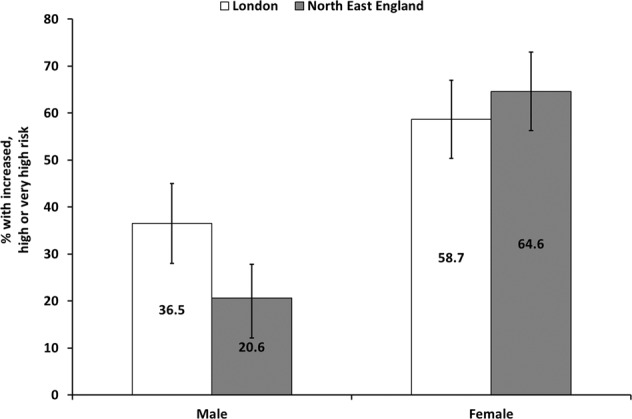
Distibution of female and male study participants according to obesity-related risk of chronic diseases, in London and North-east England (No colour bars = London, grey bars = North-east England).

Figure 1 presents the distribution of predisposition to OR-NDCs by sex. A significantly higher proportion of females than males (42 vs. 12%) had high or very high risk. Overall, the probability of developing OR-NCDs was low for 55% of participants, while 18%, 15% and 13% had moderate, high and very high probabilities, respectively. When assessed using BMI classifications, overall 8% of those with a normal BMI had an increased risk of OR-NCDs, which was much higher among those with overweight (68%) or obesity (100%) (Supplementary Fig. 1). Figure 2 shows the predisposition to OR-NCDs by sex in London and the NE. Compared with London, more females (65 vs. 59%) in the NE had risks of developing OR-NCDs.

Table 4 presents factors associated with predisposition to OR-NCDs among UK Bangladeshi migrants. The odds of developing OR-NCDs was 60% lower among those in the NE compared to London. Females had a significantly higher odds of developing OR-NCDs compared to males. However, the difference between the probability of developing OR-NCDs between males and females was three times higher in the NE than in London. Similarly, the odds of OR-NCDs was almost double among second-generation individuals compared to first-generation adult migrants. Although adjusted for age, sex and cohort, the odds for OR-NCDs was three times higher among married subjects than single ones. Current financial adversity was also associated with higher predisposition to OR-NCDs. Compared with those who considered themselves to be ‘comfortable/well off’, the odds of being predisposed to OR-NCDs was significantly higher among those reporting to be ‘okay’ (Odds ratio = 1.834, CI: 1.176, 2.859). However, OR-NCD risk did not vary significantly between those who were ‘comfortable/well off’ vs. those who were ‘struggling’. OR-NCD risk was significantly higher among those with low acculturation than those with high acculturation level. Walking ≥20 min daily was negatively associated with OR-NCD predisposition, and the odds were 32% lower for those who walked ≥20 min/day than those who walked for a shorter duration.

**Table 4:** Predisposition to OR-NCDs among 517 British-Bangladeshis in north-east and southern England using a generalised estimating equation model and sensitivity analysis using imputed data.

Variable: Categories	Odds ratio^a^ (available cases)	*p* value	Odds ratio (Imputed data)^b^	*p* value
	*n* = 517		n = 562	
Intercept	0.031 (0.015, 0.066)	<0.001	0.034 (0.011, 0.110)	0.013
Age (years)	1.045 (1.028, 1.062)	<0.001	1.047 (1.016, 1.080)	0.004
Sex: Female	2.012 (1.186, 3.413)	0.010	2.126 (1.175, 3.846)	0.013
Male	Ref		Ref	
Region: North-east	0.383 (0.281, 0.522)	<0.001	0.444 (0.258, 0.765)	0.005
South (London)	Ref		Ref	
Gender × Region: North-east (female–male)	3.288 (1.914, 5.648)	<0.001	2.891 (1.480, 5.647)	0.002
London (female–male)	Ref		Ref	
Generation: 2nd generation	2.530 (1.479, 4.326)	0.004	2.321 (1.258, 4.279)	0.007
First -generation (child)	0.893 (0.610, 1.309)	0.562	0.930 (0.620, 1.395)	0.725
First-generation (adult)	Ref		Ref	
Marital status: Married	3.013 (1.663, 5.459)	<0.001	2.515 (1.250, 5.062)	0.010
Separated/divorced	2.006 (0.712, 5.651)	0.188	1.750 (0.569, 5.376)	0.328
Single	Ref		Ref	
Current financial status: Okay	1.769 (1.130, 2.770)	0.013	1.741 (1.095, 2.769)	0.019
Struggling	1.467 (0.693, 3.109)	0.317	1.360 (0.659, 2.828)	0.410
Comfortable/well off	Ref		Ref	
Acculturation level: Low	1.711 (1.283, 2.283)	<0.001	1.635 (0.947, 2.821)	0.076
Medium	1.329 (0.956, 1.848)	0.090	1.300 (0.864, 1.956)	0.206
High	Ref		Ref	
Walking 20 min: Yes	0. 669 (0.492, 0.908)	0.011	0. 645 (0.468, 0.888)	0.007
No	Ref		Ref	

Models used exchangeable correlation structure.

^a^95% confidence intervals shown in parenthesis.

^b^Multiple imputation done as sensitivity analysis.

To check implication of missing data, we applied multiple imputation using chained equations, which resulted largely in similar results as the actual available data (Table 4). The results indicate very small differences, both in significance and magnitude of estimates between the imputed model and the original. Furthermore, the model reflected a fair predictive ability (AUC = 0.719, 95% CI: 0.675–0.763) (Supplementary Fig. 2) when evaluated using a ten-fold cross-validation.

## Discussion

This paper examined factors associated with predisposition to OR-NCDs among different generations of UK Bangladeshis living either in London or NE England. The odds of predisposition to OR-NCDs were significantly higher among females than males; among married individuals compared with single people; among subjects living in the south-east compared with those living in NE England; among British-born Bangladeshis compared to migrants; among the poorest subjects; and among those who did not walk regularly for ≥20 min. There was a noticeable sex difference, with a significantly larger difference between UK Bangladeshis in NE males and females than in Londoners.

Our findings of a relatively high prevalence of generalised obesity (females: 19%; males: 10%) and predisposition to OR-NCDS among UK Bangladeshis (females: 20% with increased risk, 42% having high or very high risk; males: 16% with increased risk, 12% with high or very high risk) conform to previous findings [3, 41]. In comparison, the Health Survey for England (HSE) has reported that BMI-based obesity prevalence increased to 17% among Bangladeshi females and 6% among males in 2004 [42]. However, BMI-based obesity prevalence tends to be lower among South Asians than estimates using WC [42]. Indeed, our study also reflected that the susceptibility of women to obesity was notably higher when abdominal obesity (WC) was used as opposed to the estimate using generalised obesity (BMI). The greater prevalence of high WC measures among Bangladeshi women has been observed by others [42], and is twice as likely compared to Black, Caribbean, Pakistani and Irish populations in the UK. Yajnik and Yudkin have pointed out that, for a given BMI, South Asians and white Europeans have different fat distributions [2]. The inadequacy of BMI in measuring fat in South Asians and the potential for underestimating the risk of OR-NCDs have been reported by several researchers [2, 36–39, 43], while studies have shown the validity of using WC over BMI in assessing NCD risks [13, 37]. Here, we found that 8% of participants with a normal BMI (18.5–24.9 kg/m^2^) had an increased likelihood of developing OR-NCDs. Similarly, Diaz et al. [38] assessed ethnic differences in prevalence of T2DM in a normal weight BMI population in both the USA and UK using the National Health and Nutrition Examination Survey and HSE data for 2003–2004. Their assessment of T2DM prevalence for those with a normal BMI among ethnic groups found that Mexican-Americans (10.9%) and Bangladeshis (10.8%) had the highest prevalence [38]. In addition, in the UK, Pakistanis (6.4%) and Bangladeshis (5.9%) had the highest prevalence (range: 1.5–6.4%) of undiagnosed T2DM. WC is a better marker of cardio-metabolic health and, since South Asians are more likely to have asymptomatic CVDs, relying solely on BMI estimates can be problematic [3, 4, 22]. Clearly, ethnic differences exist for OR-NCDs with a normal BMI, and traditional measures can underestimate the risk in South Asians [39]. The promotion of preventative strategies, for example, NHS Health Checks, can therefore be useful to reduce disproportionate disease burdens among ethnic minorities [44].

Our findings of a threefold higher predisposition to OR-NCDs among married individuals has also been observed in other countries [45–47]. In Greece, married adults had 3 and 2.5 times higher prevalence of obesity for men and women, respectively, compared to single or divorced/widowed participants [47]. Similarly, a higher prevalence among married women as opposed to men was also seen in African-Americans [46]. One study explained that single males and females maintain weight to increase their partnership prospects, whereas after finding a partner a more relaxed attitude and other social obligations dictated a more frequent intake of richer and denser foods that increased individual BMI [45]. In our sample, a higher proportion of females than males (88 vs. 71%) were married. Lifestyle factors (marriage prospects, social obligations) and physiological conditions (pregnancies, inactivity) may have contributed to these differences in OR-NCD predisposition [8, 45].

When comparing the NE and London, the lower percentages of predisposition to OR-NCDs in NE Bangladeshis may seem counterintuitive given the poorer health, productivity gap and high unemployment in this region [30]. However, results should be interpreted with caution since London is a diverse, unequal city. We recruited participants from boroughs with a higher percentage of Bangladeshis, and recent national health inequality reports suggest that Black and minority ethnic (BAME) groups tend to cluster in deprived neighbourhoods [11, 48, 49]. One report found an 11% gap in the prevalence of excess weight among adults between the most and least deprived areas of England [17]. Research has found that the life expectancy of London males living in areas with a high density of ethnic minorities is 1-year lower than white groups [49]. The same study found that ethnic minority females born in Tower Hamlets or Newham had a 5 year reduced life expectancy compared to those born in Kensington and Chelsea [49]. Analyses of UK Household Longitudinal Survey data with representative samples of ethnic groups found that BAME groups tend to live in areas with a high density of people from their own ethnic group; this was associated with poor health mediated by deprivation at the individual and area level [50]. Neighbourhood environments also influenced lifestyle choices in England. Compared to areas with a low density of Bangladeshis, significantly more fast food outlets were present in areas with higher densities. However, such associations were not seen for indoor or outdoor exercise facilities [11]. Although we found a positive association between OR-NCD risk and low acculturation, one review reported inconsistent findings [51].

We found that the predisposition to OR-NCDs was significantly higher among second-generation Bangladeshis. Neil et al. have examined inter-generational differences in health and, specifically, obesity prevalence among ethnic groups in the UK [52, 53]. Obesity prevalence was significantly higher among second-generation Indians than Europeans [53], but it was consistently higher among all ethnic minorities when acculturative changes and social mobility were adjusted. To promote healthy lifestyles and reduce OR-NCDs risk among vulnerable groups, promotion of preventative strategies [44], participatory research to gain in-depth understandings of barriers and facilitators to healthy lifestyles, community attitudes about healthy practices and testing the effectiveness of culturally sensitive interventions are needed [54].

In addition to the easy availability of cheaply priced, energy-dense and nutrient-poor foods marketed in deprived areas [11], physical inactivity worsens the health of ethnic minorities. Fewer than 10% of Bangladeshis participated in regular physical activity. Both subjective and objective studies of physical activity commonly found that, among ethnic minorities, Bangladeshi men and women had the highest levels of inactivity, and a high risk for OR-NCDs [8, 55]. Khanam and Costarelli found that Bangladeshi women were reluctant to join outdoor sports and preferred female-only swimming sessions [7]. Inactivity was even more apparent among first-generation Bangladeshi women [7, 56]. Walking for at least 20 min on a daily basis, however, significantly reduced the predisposition to OR-NCDs. A higher percentage of females than males walked for ≥20 min/day; women may walk their children regularly to school compared to men. Walking is relatively easy, even in urban environments, and also for those with reduced mobility, and could form the basis for successful exercise interventions among Bangladeshis in order to reduce their risks for OR-NCDs [57, 58].

The mediating role of SES influencing the health of ethnic minorities is reported in research examining ethnic differences in obesity prevalence [3]. OR-NCDs risk was significantly higher among those who were financially ‘okay’ compared to those who were ‘well-off’ or ‘comfortable’. However, the association of poverty and ethnic density may act differently for physical and mental wellbeing, and a higher density of own ethnic group was positively associated with mental health and wellbeing [59, 60]. Residential place matters significantly in influencing health, and is often determined by the underlying characteristics and wider socioeconomic characteristics to which residents are exposed [28].

Although this study presents some important findings, it is limited by being cross-sectional and non-probabilistic; the results presented for determinants of predisposition to OR-NCDs may therefore not be generalisable. Furthermore, due to funding restrictions we could not collect data among white Europeans or other ethnic minorities. This limited our capacity to compare prevalence estimates and determinants directly to other non-Bangladeshis. For practical reasons, our study also collected self-reported data on dietary practices and physical activity which can be less accurate than objective measures.

## Conclusion

Further research is needed to understand inter- and intra-regional health inequalities among marginalised communities and ethnic groups in the UK. However, OR-NCDs worsen the health of ethnic minorities and widen existing inequalities. It is already documented that Bangladeshis generally have higher rates of OR-NCDs than white Europeans in the UK. In our study here, Bangladeshis in London had a higher predisposition to OR-NCDs compared to those in NE England even though general measures of deprivation are higher in the NE. However, a large sex difference also existed, with a significantly higher proportion of NE females being prone to OR-NCDs. These findings reveal important inter- and intra-regional inequalities in OR-NCDs risk that need further illumination. A whole systems approach, underpinned by working with community and voluntary sectors to raise awareness of health issues, promoting preventative strategies and lifestyle choices such as walking, while also dealing with social determinants of health, are required to lower risks of morbidity among Bangladeshis. Such interventions need to be culturally sensitive and sustainable to ensure their long-term success.

## Supplementary information

Supplementary Tables and Figures
