# Supplementary material for: Risk factors for non-communicable diseases related to obesity among first- and second-generation Bangladeshi migrants living in north-east or south-east England
Source: Int J Obes (Lond). 2021 May 4;45(7):1588–98. doi: 10.1038/s41366-021-00822-5 (PMC8236404; doi:10.1038/s41366-021-00822-5)
Supplement: Supplementary file 1 — Supplementary Tables and Figures [file 41366_2021_822_MOESM1_ESM.docx]

# Supplementary materials:

**Supplementary Table 1. NICE categorisation of overweight and obesity-related health risks for chronic diseases in adults using BMI and waist circumference**

**Supplementary Figure 1. Distribution of at risk population using BMI and WC composite indicator by BMI grades**

**Supplementary Table 2. Supplementary Table 2. Bivariate associations of region, sex, and age with covariates**

**Supplementary Figure 2. Receiver operating characteristic (ROC) curve with Mean *Area Under Curve* (AUC) (CV score = 0.719, 95% confidence intervals: 0.675, 0.763) generated by 10-fold cross validation to evaluate predictive performance of the model**

# Supplementary Table 1. NICE categorisation of overweight and obesity-related health risks for chronic diseases in adults using BMI and waist circumference*

| **Body Mass Index (BMI) classification** | **Waist circumference (WC)** | | |
| --- | --- | --- | --- |
|  | **Low** | **High** | **Very high** |
| Overweight | No increased risk | Increased risk | High risk |
| Obesity | Increased risk | High risk | Very high risk |
| For men, waist circumference < 94 cm is low, 94–102 cm is high and >102 cm is very high. | | | |
| For women, waist circumference < 80 cm is low, 80–88 cm is high and >88 cm is very high | | | |

* National Centre for Clinical Excellence (NICE). Obesity: identification, assessment and management of overweight and obesity in children, young people and adults

# Supplementary Figure 1. Distribution of at risk population using BMI and WC composite indicator by BMI grades

**
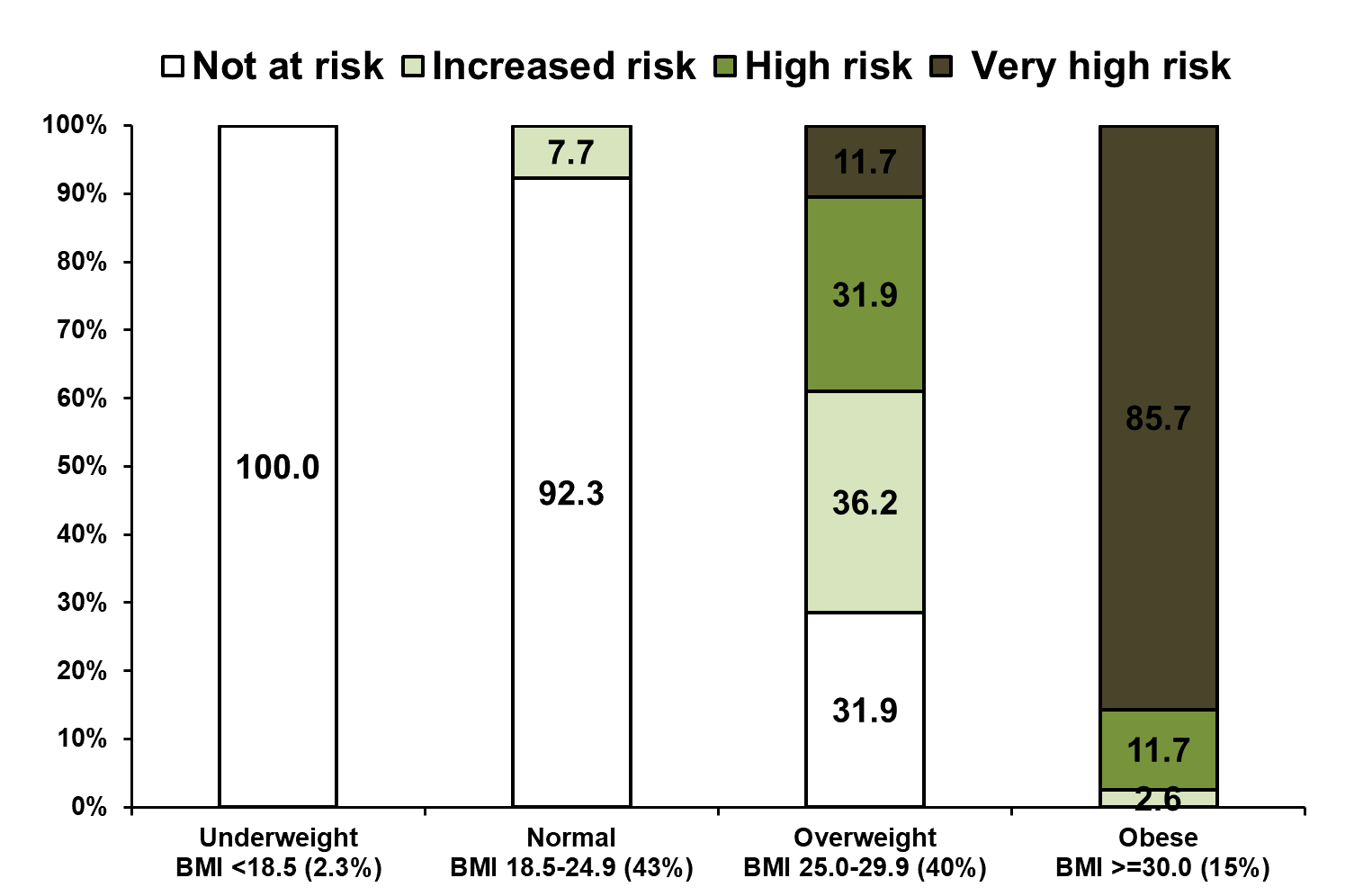
**

# Supplementary Table 2. Bivariate associations of region, sex, and age with covariates

| **Variables** | **Region ^┼^** | **Sex^┼┼^** | **Age** |
| --- | --- | --- | --- |
|  | **Odds ratio (95% CI)** | | |
| Age | 0.23 (0.06, 0.90) | 0.89 (0.57, 1.39) |  |
| Sex | 0.91(0.73, 1.13) |  | 0.99 (0.97, 1.02) |
| Generation: 2nd-generation | 4.97 (1.03, 24.03)* | 1.02 (0.76, 1.35) | 0.86 (0.81, 0.91) |
| First-generation (child) | 6.20 (1.27, 30.21)* | 1.16 (0.86, 1.56) | 1.01 (0.99, 1.04) |
| First-generation adult | Ref | Ref | Ref |
| Marital status : Separated/ divorced | 1.99 (1.15, 3.45)* | 12.70 (3.73, 43.23)* | 1.40 (1.27, 1.54)* |
| Married | 1.13 (0.75, 1.71) | 6.94 (4.68, 10.29)* | 1.35 (1.27, 1.44)* |
| Single | Ref | Ref | Ref |
| Current financial status: Okay | 1.19 (0.91, 1.55) | 1.32 (0.96, 1.82) | 1.02 (0.99, 1.06) |
| Struggling | 2.03 (1.22, 3.35)* | 1.23 (0.79, 1.91) | 1.07 (1.02, 1.12) |
| Comfortable /well off | Ref | Ref | Ref |
| Acculturation level: Low | 1.53 (0.88, 2.68) | 1.62 (1.02, 2.59)* | 1.17 (1.12, 1.21)* |
| Medium | 1.33 (1.15, 1.55)* | 1.08 (0.81, 1.46) | 1.08 (1.05, 1.11)* |
| High | Ref | Ref | Ref |
| Walking 20 minutes/day : Yes | 0.96 (0.76, 1.20) | 1.26 (1.03, 1.53)* | 1.00 (0.98, 1.04)* |
| No | Ref | Ref | Ref |

95% confidence intervals are shown in parenthesis. * denotes significant differences with *P <0.05*.

**^┼^**Region: Odds ratio reflect odds for north-east against South (London), for example: North has significantly higher odds of 2^nd^ generation than in the South.

**^┼┼^** Sex: Odds ratio reflects odds for female against odd for male, e.g. Females had significantly higher odds of separated/ divorced than that of males. Table shows unadjusted results.

# Supplementary Figure 2. Receiver operating characteristic (ROC) curve with estimated mean Area Under Curve (AUC) (CV score = 0.719, 95% confidence intervals: 0.675, 0.763) generated by 10-fold cross validation to evaluate predictive performance of the model.


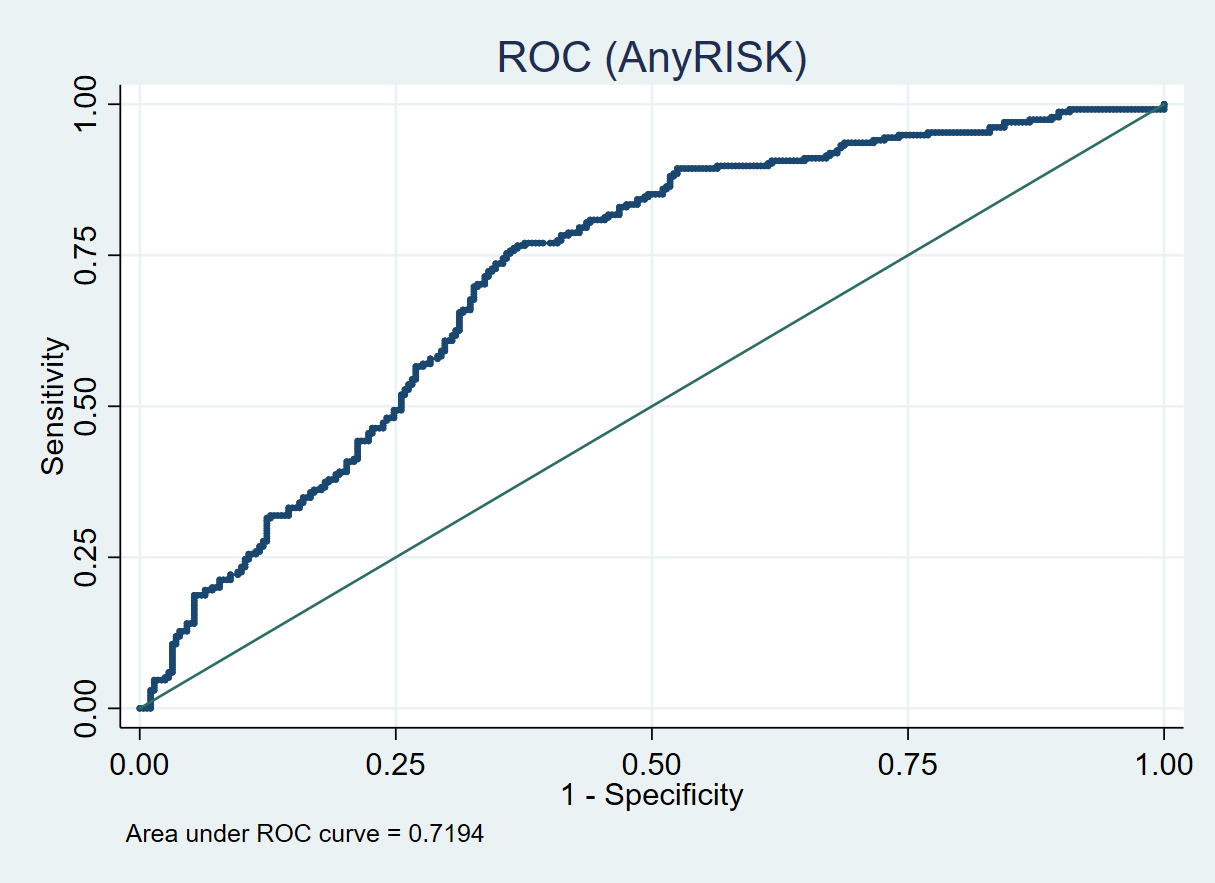


ROC curve generated using *roctab* command in STATA version 15.1
